# Early Virological and Immunological Events in Asymptomatic Epstein-Barr Virus Infection in African Children

**DOI:** 10.1371/journal.ppat.1004746

**Published:** 2015-03-27

**Authors:** Shamanthi Jayasooriya, Thushan I. de Silva, Jainaba Njie-jobe, Chilel Sanyang, Alison M. Leese, Andrew I. Bell, Karen A. McAulay, Peng Yanchun, Heather M. Long, Tao Dong, Hilton C. Whittle, Alan B. Rickinson, Sarah L. Rowland-Jones, Andrew D. Hislop, Katie L. Flanagan

**Affiliations:** 1 Medical Research Council Laboratories, Fajara, The Gambia; 2 School of Cancer Sciences, University of Birmingham, Edgbaston, United Kingdom; 3 Department of Infection and Immunity, The University of Sheffield Medical School, Sheffield, United Kingdom; 4 The Weatherall Institute of Molecular Medicine, University of Oxford, John Radcliffe Hospital, Headington, Oxford, United Kingdom; 5 MRC-University of Glasgow Centre for Virus Research, Institute of Infection, Immunity and Inflammation, University of Glasgow, Glasgow, United Kingdom; 6 London School of Hygiene and Tropical Medicine, London, United Kingdom; 7 Nuffied Department of Medicine, NDM Research Building, University of Oxford, Old Road Campus, Headington, United Kingdom; 8 Department of Immunology, Monash University, Commercial Road, Prahran, Melbourne, Victoria, Australia; State University of New York Upstate Medical University, UNITED STATES

## Abstract

Epstein-Barr virus (EBV) infection often occurs in early childhood and is asymptomatic. However, if delayed until adolescence, primary infection may manifest as acute infectious mononucleosis (AIM), a febrile illness characterised by global CD8+ T-cell lymphocytosis, much of it reflecting a huge expansion of activated EBV-specific CD8+ T-cells. While the events of AIM have been intensely studied, little is known about how these relate to asymptomatic primary infection. Here Gambian children (14–18 months old, an age at which many acquire the virus) were followed for the ensuing six months, monitoring circulating EBV loads, antibody status against virus capsid antigen (VCA) and both total and virus-specific CD8+ T-cell numbers. Many children were IgG anti-VCA-positive and, though no longer IgM-positive, still retained high virus loads comparable to AIM patients and had detectable EBV-specific T-cells, some still expressing activation markers. Virus loads and the frequency/activation status of specific T-cells decreased over time, consistent with resolution of a relatively recent primary infection. Six children with similarly high EBV loads were IgM anti-VCA-positive, indicating very recent infection. In three of these donors with HLA types allowing MHC-tetramer analysis, highly activated EBV-specific T-cells were detectable in the blood with one individual epitope response reaching 15% of all CD8+ T-cells. That response was culled and the cells lost activation markers over time, just as seen in AIM. However, unlike AIM, these events occurred without marked expansion of total CD8+ numbers. Thus asymptomatic EBV infection in children elicits a virus-specific CD8+ T-cell response that can control the infection without over-expansion; conversely, in AIM it appears the CD8 over-expansion, rather than virus load per se, is the cause of disease symptoms.

## Author Summary

Primary infection with EBV, a common human herpesvirus, is typically asymptomatic in childhood but, if occurring in adolescence or later, often presents as AIM. This febrile illness is characterised by high virus loads in the blood and an exaggerated EBV-specific CD8+ T-cell response that pushes total CD8+ T-cell numbers well above normal levels. By contrast, very little is known about the events of asymptomatic primary infection. We therefore studied young Gambian children at an age at which many acquire EBV, monitoring them over six months for evidence of EBV infection by virus load in the blood, virus-specific IgM and IgG antibody status, and virus-specific CD8+ T-cell responses. Focusing on IgM-positive children with very recent EBV infection but no history of symptoms, we found that they carried a virus load equivalent to that seen in AIM patients and also mounted a classical virus-specific CD8+ T-cell response. However, that response, though it could occupy at least 15% of the circulating CD8+ T-cell pool, occurred without the huge global expansion of CD8 numbers seen in AIM. This work reinforces the idea that the host’s exaggerated CD8+ T-cell response, rather than the virus load per se, leads to the symptoms of AIM.

## Introduction

Epstein-Barr Virus (EBV) is a ubiquitous gamma herpesvirus associated with occasional severe primary infections, several malignancies and significant pathology in immunosuppressed hosts. It does not, however, cause significant morbidity in the majority of those infected. In The Gambia most children are infected during childhood, in contrast to most developed countries where the majority of primary infections occur at a later age, often in adolescence [1,2]. It is estimated that between a quarter and up to three quarters of those infected in adolescence will develop a sometimes-severe disease, AIM [3,4]. Paradoxically, those infected during childhood tend to have minor self-limiting illnesses that often go undetected [5]. It is not fully understood why individuals that contract EBV during childhood are usually asymptomatic and do not develop AIM. Of note, most of the published literature regarding the immunopathogenesis of primary EBV infection is derived from studies of AIM, rather than asymptomatic infections.

Many studies in adults have characterised cellular immune responses *ex vivo* during AIM, both among CD8+ and to a lesser extent CD4+ T-cell subsets [6–13]. The EBV-specific CD8+ T-cell response is hugely amplified, such that total CD8+ T-cell numbers in the blood may reach five to ten-fold higher than usual. Indeed individual lytic antigen reactivities (typically against epitopes within the immediate early (IE) and some early (E) proteins) can account for up to 40% of the highly expanded CD8+ T-cell population, and individual latent antigen reactivities (typically against epitopes from the EBV nuclear antigen 3A, 3B, 3C family) occupying up to 5%. These CD8+ T-cells display a phenotype consistent with recent antigen stimulation, being perforin-positive with direct *ex vivo* cytotoxic function [14–16] and express the activation marker CD38 and cell cycling marker Ki-67 [8,10,14,17]. What drives these expansions in AIM is unclear, but factors such as an initial lack of natural killer cell control [18], cross-reactive recognition by clonotypes in pre-existing CD8+ T-cell memory [19], and genetic factors [20–22], including polymorphism of the IL-10 promoter [23], have all been proposed.

Whether the cellular response to early EBV infection in asymptomatic children shows features of disruption similar to those described in AIM has been difficult to investigate, mostly because donors without clinical symptoms can’t be readily identified. However, an understanding of how EBV infection is controlled with minimal immunopathology, i.e. without the development of AIM, is important, as AIM is associated with an increased risk of EBV-associated diseases such as EBV-positive Hodgkin lymphoma and multiple sclerosis [24,25]. Of the few published studies on asymptomatic primary EBV infections, Silins et al identified four adult patients undergoing silent seroconversion within a vaccine trial [26]. Interestingly some of these had high EBV loads yet did not have massive T-cell expansions and, where studied, most did not have the distorted T-cell receptor (TcR) repertoires usually seen in AIM; however, their EBV-specific T-cell response was not studied. As to infections during childhood, an early report suggested that this occurs with a serological picture distinct from AIM and without obvious lymphocytosis [27], while another small study of children aged 20–35 months detected EBNA3A, B and C-specific CD8+ T-cell responses in the blood without addressing issues of viral load or hyper-expansion [28].

A detailed study of the EBV-specific immune response and EBV dynamics in asymptomatically infected children utilising modern immunological and virological tools is lacking. The present work follows a cohort of 114 Gambian children longitudinally over six months, using serology and viral load to determine EBV status. It describes the EBV-specific CD8+ T cell responses in those that have had a relatively recent primary EBV infection without any obvious clinical history, and additionally captures six children undergoing silent seroconversion.

## Results

### Demographics and EBV serostatus of Gambian children

Gambian children of an age, 14–18 months, likely to be undergoing primary EBV infection were recruited for study from an infant vaccination clinic. Blood samples were collected from the children at baseline (visit one), they were vaccinated one week later against Diptheria, Tetanus, Pertussis, Hepatitis B and *Haemophilus influenzae B* (visit two). A five-millilitre blood sample was collected a week later (visit three) primarily to monitor vaccine responses and a further sample at six months (visit four). Of 120 children screened, six were ineligible due to concurrent illnesses or malnutrition at screening whereas 114 were enrolled, of which 99 remained in the study until completion at six months. The study dropouts did not significantly differ in age, sex, weight, haemoglobin, leucocyte and lymphocyte counts, compared to those who continued to participate (S1 Table).

**Fig 1:**
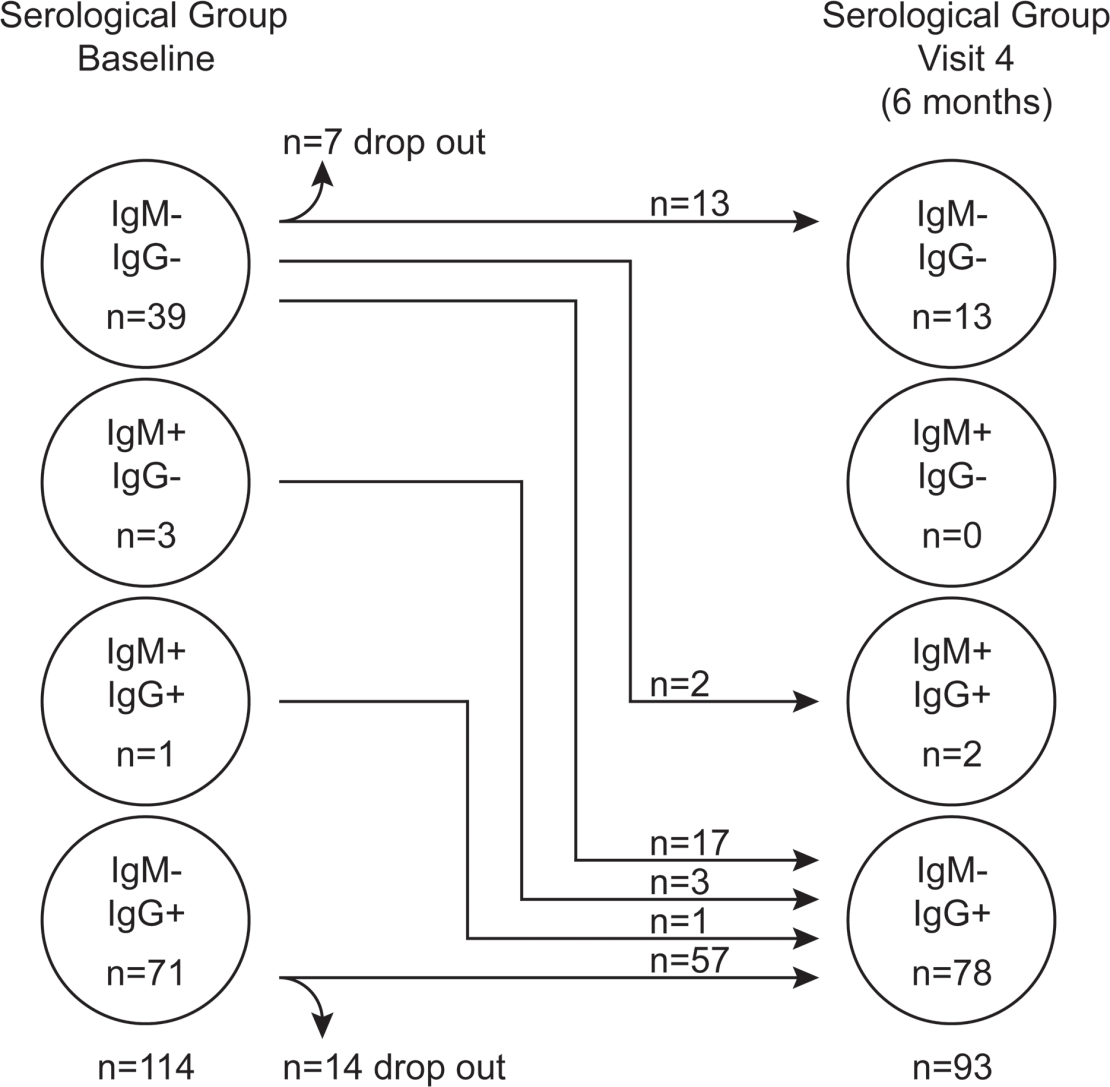
EBV VCA serological status of Gambian children. Serological status of study participants at baseline (visit one) and six months later at visit four.

Initially children were tested for their EBV-VCA antibody status and categorised into one of three groups: non-infected (IgM−IgG−), established infection (IgM−IgG+) or very recently infected (IgM+IgG+/−). At visit one, 71 children had established EBV infection as judged by the presence of VCA-specific antibodies of IgG but not IgM class (Fig. 1). Another four children showed evidence of recent infection, with three having IgM VCA-specific antibodies only and one having both IgM and IgG VCA-specific antibodies. The remaining 39 children appeared to be non-infected, having no detectable VCA-specific antibodies or viral genomes in their peripheral blood mononuclear cells (PBMCs). At visit four (six months later) 17 of these 39 initially EBV non-infected donors had become VCA IgM−IgG+, another two had become VCA IgM+IgG+, 13 remained VCA IgM−IgG−, while seven dropped out of the study. The four initially IgM+ children had now become IgM- and had developed VCA-specific IgG. All children, including those with VCA-specific IgM antibodies, were asymptomatic for classical symptoms of AIM (fever, lymphadenopathy, malaise) prior to recruitment and at subsequent visits, based on maternal history and clinical evaluation. Overall, 62% of children showed serological evidence of being EBV infected at baseline, rising to 86% among those remaining in the study six months later. An analysis of VCA IgG titre in a subset of 25 pairs of samples from children at visit one and four showed no significant difference in titre (p = 0.774, S1A Fig).

### Recently-infected asymptomatic Gambian children have EBV genome loads equivalent to donors with acute infectious mononucleosis

**Fig 2:**
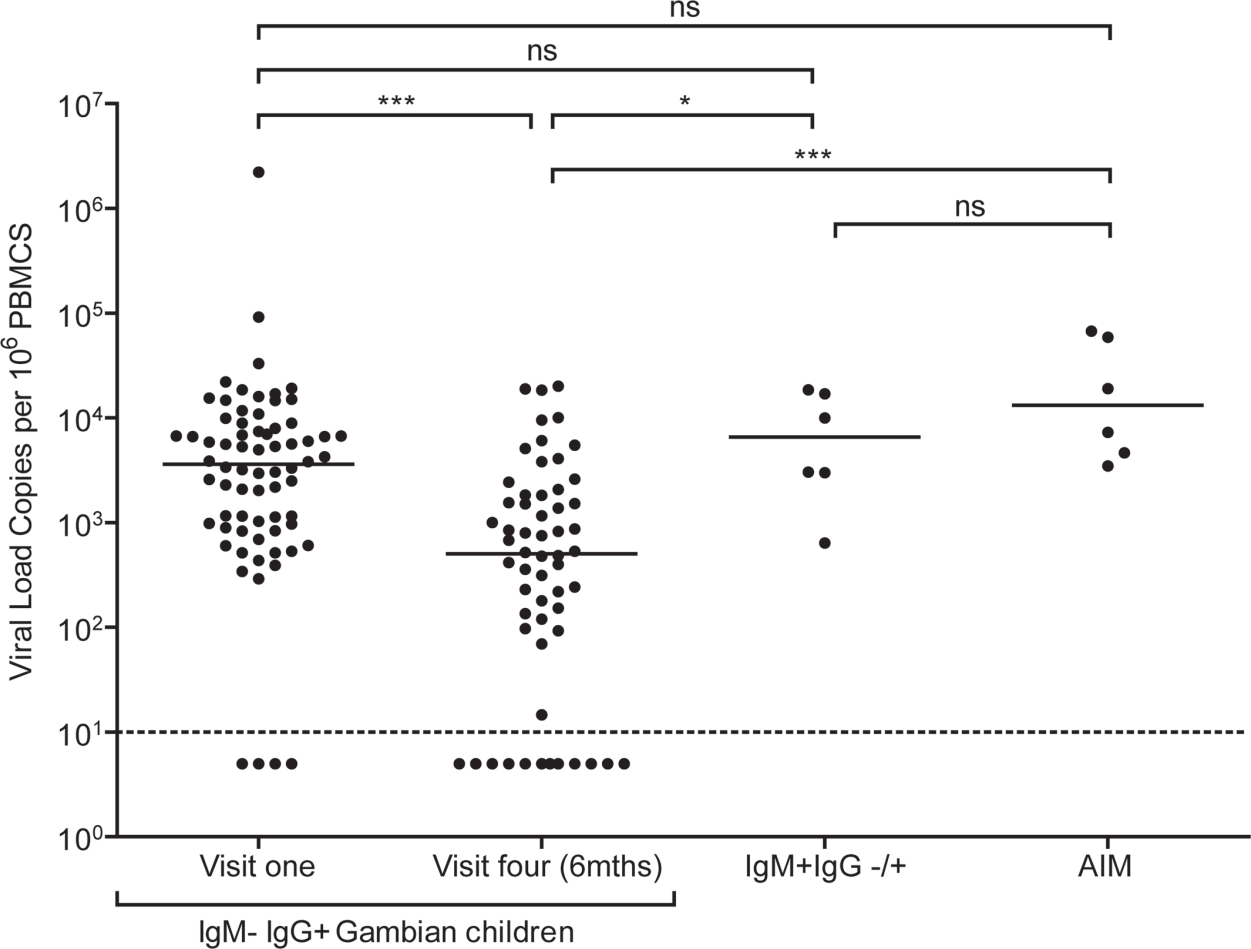
EBV loads in peripheral blood mononuclear cells (PBMCs) from Gambian infants compared to Acute Infectious Mononuclear (AIM) patients. EBV genome loads in IgM-IgG+ Gambian children at visit one (n = 70) and after six months at visit four (n = 58), compared to IgM+IgG+/- children from either time point. For comparison, data of virus loads measured in Caucasian adolescent AIM patients are also presented. The dashed line represents the lower limit of detection for EBV genomes in the assay. Donors below the dashed line had undetectable viral loads. *P* values calculated using Dunn’s Multiple Comparison Test (one way analysis of variance (ANOVA)). * p<0.05 ** P<0.01 *** P<0.001

Since acquisition of EBV in similar African cohorts begins between six and twelve months after birth [29,30], it is likely that at least some children who were IgG reactive to VCA within the cohort were infected with EBV within the last six months prior to recruitment. To examine for evidence of recent infection among these donors, EBV genome loads in PBMCs were measured by qPCR analysis. Fig. 2 shows viral genome load data from PBMCs collected from 70 IgM−IgG+ donors at baseline, 58 of these donors six months later, and six very recently infected IgM VCA reactive donors (some of whom also had VCA-specific IgG antibodies). Genome load data from Caucasian adolescent patients undergoing primary symptomatic EBV infection, AIM, assessed using the same qPCR assay are also included for comparison.

Almost all of the children who had IgG antibodies to VCA at baseline had high EBV genome levels in their PBMCs, ranging up to two million genomes per million PBMCs. Indeed, for the whole cohort of IgM− IgG+ children sampled at visit 1, the median load of 3000 genomes per million PBMCs (IQR 900 to 8000 genomes per million PBMCs) was not significantly different to that observed in AIM patients. When PBMCs from these same children were assessed for genome loads six months later, a narrower range of values was found and the median load was eight to tenfold lower than at baseline. The decreasing virus loads observed over these two time points suggests that these donors were establishing their virus host balance following recent EBV infection. Comparing virus load to VCA IgG titre in a subset of 25 children at the two time points showed no correlation between load and titre (S1B and C Fig). When the samples from the six IgM+ donors were analysed, these also showed high EBV genome loads with a median value of 8000 genomes per million PBMC, slightly higher than but not significantly different from loads in IgM-IgG+ positive children measured at baseline and again similar to that seen in AIM patients. Such data are consistent with these IgM+ children having been very recently EBV-infected.

### Lymphocyte counts are not altered in asymptomatic primary EBV infection in Gambian children

**Fig 3:**
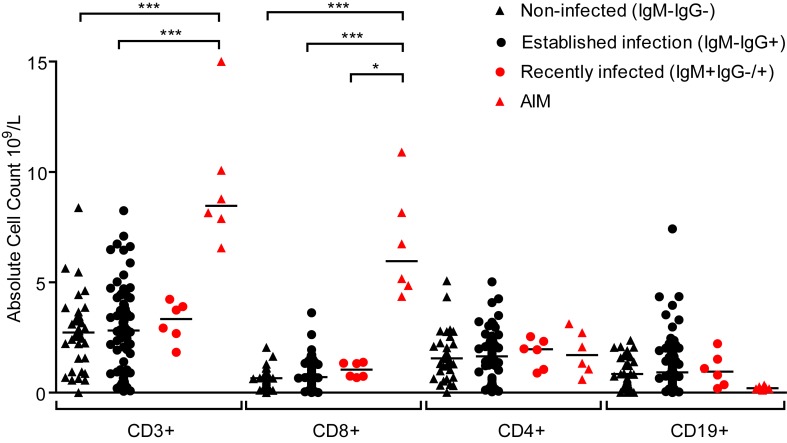
Size of lymphocyte populations in the blood of Gambian children and AIM patients. Absolute numbers of selected T and B-cell subsets were measured from EBV non-infected (IgM-IgG-), established infection (IgM-IgG+) or recently infected (IgM+IgG+/-) Gambian children and UK donors with AIM. Counts were based on full blood count analysis to obtain lymphocyte numbers and flow cytometric analysis to identify population frequencies. No significant differences in subset counts were observed between different donor groups in Gambian children. UK IM donors had a significantly greater proportion CD8+ T-cells. *P* values calculated using Dunn’s Multiple Comparison Test (one way analysis of variance (ANOVA)). * p<0.05 ** P<0.01 *** P<0.001.

Primary symptomatic infection with EBV is associated with dramatic expansions in the frequency and absolute number of lymphocyte subsets, especially CD8+ lymphocytes [6]. Evidence of disruptions to the lymphocyte compartments of the three groups of children (IgM− IgG−, IgM− IgG+ or IgM+ IgG+/−) were studied by determining absolute numbers of lymphocytes within the CD3, CD4, CD8 and CD19 subsets. Fig. 3 shows results of absolute cell counts and, for comparison, counts of equivalent subsets from six Caucasian AIM patients. Dramatic expansions of the CD3+ and CD8+ (but not CD4+) T-cell numbers and a contraction of B cell numbers were seen in AIM patient samples. However, no obvious or significant expansion of lymphocyte subsets was observed when comparing uninfected children with the two EBV-infected groups. Furthermore, no significant changes in the CD4:CD8 ratios were observed in PBMCs from a subset of 14 children over the six month study period (p = 0.76, S2 Fig). This indicated that there was little disruption to peripheral lymphocyte subsets in children at these different stages of asymptomatic EBV infection.

### EBV-specific CD8+ T-cells from children with established EBV infection show evidence of recent activation

**Fig 4:**
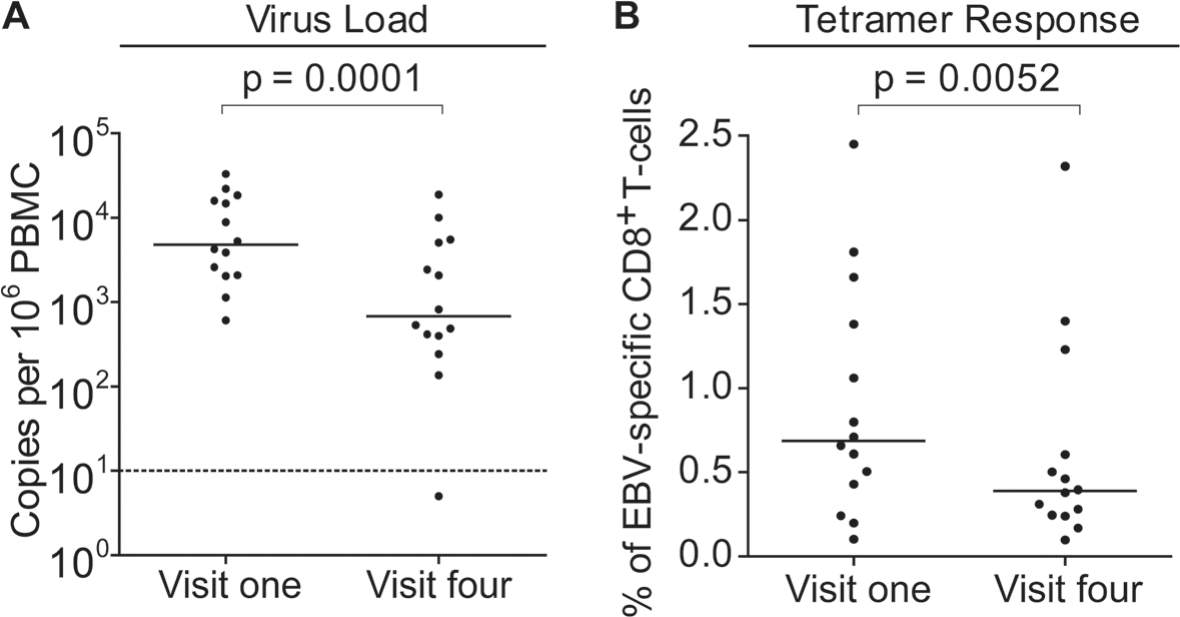
EBV genome loads and EBV-specific T-cell responses in IgM-IgG+ Gambian infants likely to have been infected several months prior to visit one. PBMCs collected from fourteen donors at visit one and visit four were analysed for (A) genome load by qPCR and (B) EBV-specific CD8+ T-cell responses by staining with HLA Class I tetramers followed by flow cytometry analysis. Results are expressed as genomes per million PBMC or % of EBV-specific T-cells among all CD8 T-cells respectively. *P* values calculated using Mann-Whitney U test.

To further understand the dynamics of CD8+ T-cell responses and virus loads at early stages of infection, MHC-class I tetramers were used to assess the frequency of EBV-specific T-cells in the PBMCs of children with VCA-specific IgM− IgG+ antibodies at baseline and six months later and compared to their virus loads at these time points. Children with relevant EBV-specific responses were identified by screening PBMCs from visit one for responses by ELISpot to pools of peptides containing peptide-epitopes known to be presented by HLA types frequent within the Gambian population. This identified 14 children with responses which could be assessed with HLA-A*0201, HLA-B*0801 or HLA-B*3501MHC class I tetramers. Virus loads from this subset of donors were representative of the overall population shown in Fig. 2 at the two time points and their loads were significantly decreased at the second time point (Fig. 4A).

Frequencies of EBV-specific responses in each of the children’s paired visit one and visit four PBMCs samples were obtained by staining with an appropriate tetramer which would identify an immunodominant response. These tetramers presented epitopes derived from the immediate early lytic cycle protein BZLF1 protein, either the HLA-B*0801 presented RAKFKQLL peptide or the B*3501 presented EPLPQGQLTAY peptide, or from the early lytic cycle protein BMLF1, the HLA-A*0201 presented GLCTLVAML peptide. Analysis of the frequency of tetramer-specific CD8+ T-cells in PBMCs from this group is shown in Fig. 4B. This shows that at the first time point EBV-specific responses are made, but their frequency of up to 2.5% of CD8+ T cells is not obviously increased as is seen in AIM patients, in whom previous reports have documented up to 40% of the total CD8+ T-cell population as being EBV-specific (Callan et al. 1998). Analysis of the visit four time point, collected six months later, showed that responses were still present, however they were on average significantly lower than those detected at visit one.

**Fig 5:**
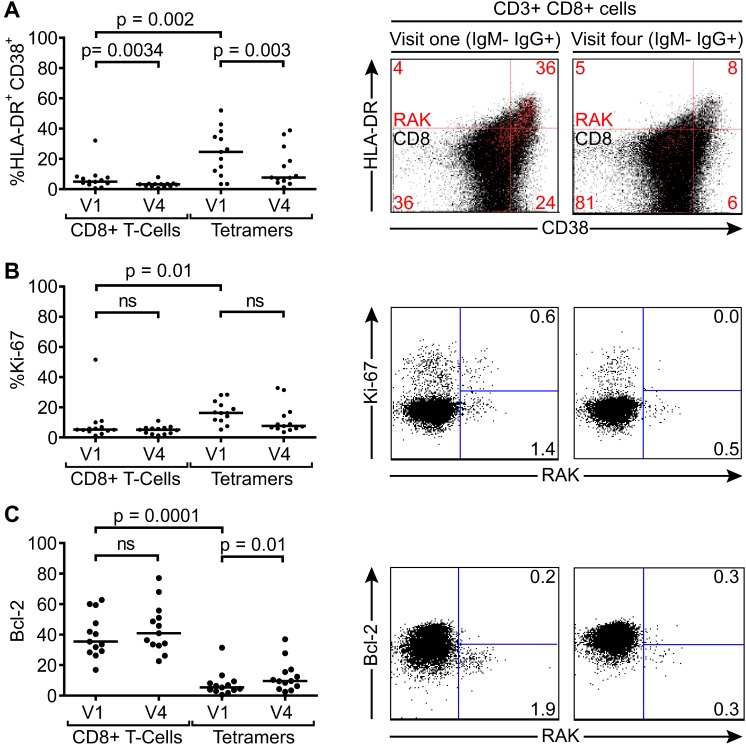
Activation, proliferation and Bcl-2 status of total and EBV-specific CD8+ T-cells in VCA IgM-IgG+ EBV infected Gambian children. Flow cytometry analysis of PMBC samples collected from children at visit one and visit four examined for co-expression of CD38 and HLADR (A), expression of intracellular Ki67 (B) and expression of Bcl-2 (C) either among the total CD8+ T-cells or the tetramer positive EBV-specific CD8+ T-cell population. Left hand graphs represent summaries of the samples studied with the y-axis indicating percentage of marker positive cells. The middle and right hand columns show representative flow cytometry analysis dot plots from representative donors illustrating the expression of markers, HLADR, CD38, Ki-67 and Bcl-2, on MHC class I tetramer positive cells at baseline and six months later. Note CD38 and HLADR flow plots are presented with data from gated tetramer positive cells (red) overlaid on total CD8+ T cells (black). *P* values calculated using Dunn’s Multiple Comparison Test (one way analysis of variance (ANOVA)).

This reduction in EBV-specific T-cell numbers with time, coupled with a falling virus load illustrates a pattern consistent with recent EBV infection and establishment of a long term carrier state in these children. In all but one of these 14 children there were sufficient cell numbers to examine the changing phenotype of the T-cell response over time. Both total CD8+ T-cells and tetramer-positive CD8+ T-cells were examined for markers known to be expressed by CD8+ T-cells responding to acute EBV infection; namely activation status as defined by co-expression of CD38 and HLA-DR, cycling status as defined by the expression of Ki-67, and apoptosis sensitivity as indicated by loss of Bcl-2, which is down regulated in activated EBV-specific cells in AIM patients. Of note CD38 and HLA-DR co-staining for activation status was used as lymphocytes from young children may constitutively express CD38 which is progressively lost with age [31]. The graphs on the left hand side of Fig. 5 summarises the results of this analysis while the flow cytometry plots show representative analyses for each stain at the two time points; note that the top flow plots combine data from all CD8+ T cells in black, with the tetramer-positive population in red, while the middle and bottom flow plots show the Ki-67/tetramer and Bcl-2/tetramer profiles. At the first time point there were significantly more HLADR+CD38+ (p = 0.002) and Ki-67+ (p = 0.01) EBV-specific CD8+ T-cells compared to the total CD8+ T-cell population, while tetramer positive CD8+ T-cells expressed lower levels of the anti-apoptotic marker Bcl-2 (p <0.0001). The percentage of HLADR+CD38+ EBV-specific CD8+ T-cells in the IgM−IgG+ children declined significantly over time (p = 0.003), whereas cellular expression of Bcl-2 significantly increased (p = 0.013), with a non-significant decline seen in frequency of cells expressing Ki-67. Again, the phenotypic analysis of these VCA antibody IgG+ donors suggests recent EBV infection and the establishment of a long term carrier state.

### Asymptomatic primary EBV-infected Gambian children lack a significant expansion of global T-cell numbers despite substantial activation of EBV-specific CD8+ T-cells

To get the clearest understanding of the early events in EBV-specific CD8+ T-cell responses in children undergoing asymptomatic infection, the EBV tetramer-specific responses and phenotype of these cells were examined in children with IgM VCA-specific antibodies. Of the six IgM+ children found in this study, three were suitable for study with the tetramer panel for frequency and phenotype analysis at the different time points. However the three other donors did not show expanded numbers of CD8+ T cells (Fig. 3) and showed, at most, small changes in the frequencies of CD8+ T cells from 40.9%, 36.8% and 50% at visit one when they were IgM+, to 27.3%, 32.5% and 50.1% at visit four respectively when they were IgG+.

**Fig 6:**
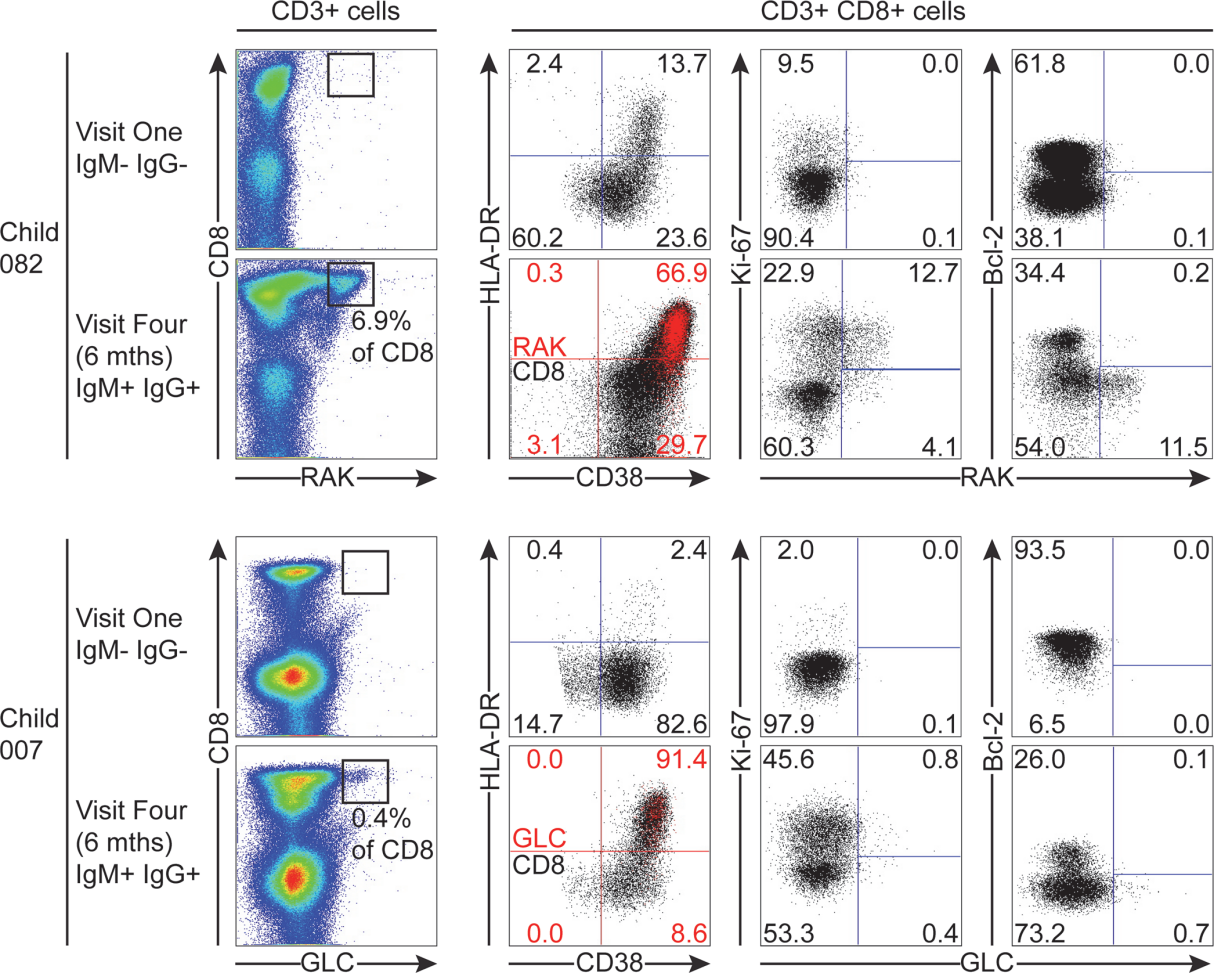
EBV-specific CD8+ T cell response and phenotype of PBMCs from children seronegative at visit one who at visit four had been very recently infected (IgM+ IgG+/-). PBMC samples from two donors that were EBV non-infected at visit one and became VCA IgM+ six months later were analysed for EBV-specific responses using appropriate MHC class I tetramers. Epitope-specific CD8+ T cells were further analysed for activation status by measuring CD38 HLA DR co-expression, cell cycle status by measuring Ki-67 status and Bcl-2 status. Flow plots and gating are presented as in Fig. 5.

Of the samples from children which could be analysed with tetramers, 082 and 007 were seronegative at the first time point, but had developed IgM+ VCA-specific antibodies at six months. Although there was no significant increase in the absolute numbers of CD8+ T-cells before and after EBV acquisition, both donors 082 and 007 showed increases in the frequencies of CD3+ CD8+ lymphocytes from baseline values of 18.2% and 19.8% to values of 50.3% and 40.8% respectively six months later. As shown in Fig. 6, tetramer analysis of samples at baseline when the donors 082 and 007 were VCA seronegative showed no tetramer-specific staining. However at six months, when the children were VCA IgM+ IgG+, the HLA-B*0801 donor 082 made a substantial response with 6.9% of their CD8+ T-cells being specific to the RAK-epitope while the HLA*0201 donor 007 made a small response of 0.43% CD8+ T-cells to the GLC-epitope. In both cases, tetramer positive cells were highly activated with the majority of EBV-specific cells co-expressing HLA-DR and CD38. In both of these donors a substantial frequency of EBV-specific cells were in cycle; interestingly in the case of child 007 a large proportion of non-tetramer-specific CD8+ T-cells were also in cycle, likely representing other EBV-specificities, possible bystander activation or coincident infection with another pathogen. In the EBV-specific CD8+ T-cells from these children there was little if any expression of Bcl-2 (Fig. 6).

**Fig 7:**
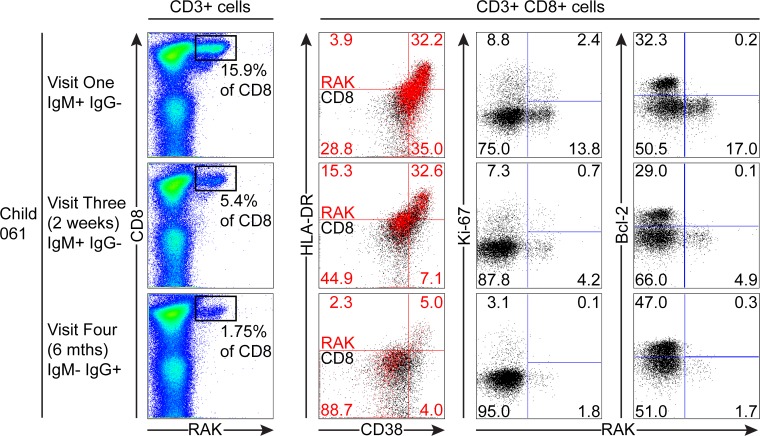
Prospective analysis of EBV-specific CD8+ T cell response and phenotype of PBMCs from a child very recently infected (IgM+ IgG−) at visit one. Serial PBMC samples from an HLA B*0801 donor found to be EBV VCA IgM+ at visit one were analysed for EBV-specific responses using the B*0801 RAK-specific MHC class I tetramer. A sample from visit three was available in addition to the one from visit four for this donor. The epitope-specific CD8+ T cells were further analysed for activation status by measuring CD38 HLA DR co-expression, cell cycle status by measuring Ki-67 status and Bcl-2 status. Flow plots and gating are presented as in Fig. 5.

The third child studied, the HLA-B*0801 donor 061, had VCA-specific IgM+ antibodies at the first time point and made a substantial response to the RAK epitope which allowed serial monitoring of this response (Fig. 7). The frequency of PBMC CD8+ T cells did not change over time with levels being 38% at visit one, 37% at three two weeks later, and 37% at visit four with no significant expansion of absolute numbers (see Fig. 1). However, as shown in Fig. 7, tetramer analysis at the first time point detected a large RAK-specific response of 15.9% of CD8+ T-cells, with 32.2% of these being activated, 14.8% in cycle and few expressing Bcl-2. After two weeks the tetramer-specific frequency had decreased to 5.4% of CD8+ T-cells with no associated decrease in activation marker and Ki-67 expression, nor increase in Bcl-2 expression by the tetramer-specific cells. By six months, the frequency of RAK-specific cells had decreased to 1.75% of CD8+ T-cells. Here the RAK-specific cells phenotypically resembled the EBV-specific cells from the VCA-IgG+ donors at visit 4, with only 5% expressing activation markers, 5% of cells in cycle and 15% expressing Bcl-2 (Fig. 7). Overall these findings suggest that in asymptomatic primary EBV infection, the frequency of activated EBV-specific cells in the CD8 population can be substantial but this occurs without significant expansion of the CD8 compartment as a whole.

## Discussion

Current understanding of the immunological changes seen during primary EBV infection is almost exclusively derived from studies of AIM in adolescents and adults. These may not apply to the situation in asymptomatic primary EBV in early childhood, when a large proportion of infections occur. In this study, virological and immunological parameters of African children, studied during a time period when they undergo primary asymptomatic EBV infection, were examined to better understand the pathogenesis of primary asymptomatic EBV infection. Children who had EBV infection established for at least some months, as judged by the presence of VCA IgG antibodies but not IgM, have high virus loads, comparable to AIM patients, and their EBV-specific CD8+ T-cells show evidence of recent activation. These loads dropped significantly when tested six months later but were still elevated when compared to loads in other populations such as UK carriers, but were similar to those detected in healthy older children from The Gambia [32]. This suggests that in this situation, the virus set point is established several months after primary infection and that it is higher than in EBV-infected carriers in the UK.

What determines the high virus set point detected in these children is unclear. One factor known to increase EBV loads is exposure to malaria, with children living in malaria endemic regions have higher EBV loads compared to those living in areas of sporadic transmission [32,33]. How EBV loads in children in the present study relate to loads in other African childhood cohorts is difficult to determine due to differences in assays and sample sources used to quantify EBV loads [33,34]. Malaria infection is thought to increase EBV loads either through promoting B cell proliferation [35] or altering T cell responses [36]. However the children in this study showed no evidence of acute malaria and data at the time of the study demonstrated a low prevalence of malaria in The Gambia [37,38]. Others have suggested virus load may be related to the age at infection, with African infants infected shortly after loss of maternal antibodies having higher virus loads than those infected later [34]. In this context, comparing virus loads to western populations should be interpreted with some caution as the timing of infection in these latter populations is relatively delayed and is particularly dependent on the ethnic group studied [39].

A clearer picture of the early immune response dynamics comes from studying children with IgM+ VCA-specific antibodies, who are likely to have had very recent EBV infection. Although these children had high virus loads in their PBMCs, akin to those seen in studies of symptomatic AIM [2,3,40], they showed no obvious physical signs of infection. There was no significant expansion of lymphocytes or the CD3+ or CD8+ T-cell compartment; a finding consistent with those of others who have studied lymphocyte compartments in primary asymptomatic EBV infections [1,26,27]. Nevertheless, there was an ongoing virus-specific CD8+ T cell response in the IgM+ children with, in one case, greater than 15% of CD8+ T cells directed against a single dominant EBV epitope. This increased frequency of EBV-specific T cells found in the periphery in the presence of high virus loads may call in to question their role in control of the infection. However we have previously found that in AIM patients EBV-specific T cells lack expression of lymphoid homing receptors such as CCR7 and CD62L which allow access to tissues such as the tonsil. Virus replication and transformation of B lymphocytes, in AIM patients at least, occurs in this tissue and appears poorly controlled by the inefficient recruitment of EBV-specific T cells to this site [10]. As activated EBV-specific T cells express low levels of CCR7 and CD62L, we propose that the activated EBV-specific T cells in the children are similarly inefficiently recruited to the tonsil and virus replication and transformation poorly controlled at this site, allowing higher loads of virus to be detected in the presence of these strong responses.

The phenotype of the antigen-specific CD8+ T-cells from the IgM+ VCA antibody children was consistent with what has been described in AIM donors, being highly activated (HLADR+CD38+), in cycle (Ki-67+) and pro-apoptotic (low Bcl-2 expression) [8,10,14,17,41,42]. Why these children do not develop the expanded numbers of CD8+ T-cells observed in AIM patients after EBV infection is not clear. Possible reasons for the AIM associated hyper-expansion have included the development of heterologous immunity where an existing response to an epitope coded by a previously encountered pathogen cross reacts with another from EBV, amplifying the pool of T-cells responsive to EBV challenge and potentially inducing an exaggerated response. Children with less antigenic exposure would have a more limited repertoire of T-cells capable of responding in this heterologous manner [19]. Alternatively two recent studies comparing the incidence of AIM in monozygotic compared to dizygotic twins, and first-, second- or third-degree relatives have shown concordance in the development of AIM, suggesting that there may be a genetic component underlying disease development [20,22]. Perhaps more importantly, an emerging concept in the control of early EBV infection comes from studies using immunodeficient mice reconstituted with human haematopoietic cells, which repopulate human NK and T-cell repertoires. Depletion of NK cells in this model followed by challenge with EBV recapitulates an AIM-like response including splenomegaly, increased plasma levels of the pro-inflammatory cytokine IFN-γ and increased CD8+ T-cell numbers and frequencies [18]. Currently there is a lack of clarity in the literature as to the dynamics and role of NK cell responses in AIM and so the immediate relevance of these experimental findings to natural infection remain to be resolved. Some studies have indicated that there are inverse relationships between NK cell numbers and both severity of symptoms and virus load [43], while others have shown a positive correlation between NK cell numbers both with virus load and severity of symptoms in AIM patients [3]. However NK cells are a heterogeneous population and so a key question arising from these studies is whether there is a difference in subsets of NK cells in terms of numbers or function between individuals who go on to develop AIM compared to children or others who don’t develop this disease.

It is important to recognise that the immune system of children is different in comparison to adolescents where AIM is typically seen. Neonates are born with high levels of the immunosuppressive cytokine IL-10, high levels of plasma immunosupressive factors such as adenosine, and have Th2 and Th17 skewed immunity, all of which decline by 1–2 years of age to near adult levels [44]. This “infant-adapted” immune profile evolves in the first few years of life, with a gradual increase in Th1 capacity, maturing of B cell and antibody responses, and development of T and B cell immunological memory [44]. By the time infants were recruited into the study at 14–18 months of age, they would have a considerably matured immune system compared to birth but some differences would persist compared to adolescents. Such immunological differences could have contributed to the asymptomatic primary infection that is seen in the children compared to adolescents.

Throughout this study we have used the VCA serological status as a guide to when infection occurred, consequently the precise timing of the primary EBV infection cannot be determined. However, from previous data on infants at the same study site, only 18% of infants were found to be EBV infected at nine months of age [29] and one can, therefore, assume that the majority of children would have been infected six months to one year prior to recruitment. Furthermore, the IgM+IgG± donors were likely infected within the last 120 days and may be at a different stage of infection. Secondly, ensuring children are truly asymptomatic in this setting can be challenging as reliance on maternal perception may not be reliable and careful clinical studies of infants or children have described AIM like symptoms in some instances [45]. To combat this, the children all underwent a health screen by the study clinician, including baseline clinical observations such as weight, height, temperature and heart rate and where indicated a rapid malaria test. Recent work by Balfour et al. has suggested that 89% of primary EBV infections in a cohort of University students display some symptoms, although they may not fulfil all classic criteria for AIM, which is different to our observations in Gambian children [3].

In summary, this study supports the notion that AIM is an immunopathological disease and that symptoms are caused by the significantly expanded CD8+ T-cell responses to the virus. It provides clear evidence that during primary asymptomatic infection EBV-specific responses are indeed activated and can occupy a significant percentage of the circulating CD8+ T cell pool. However these responses appear able to contain the infection without the massive expansion that characterises AIM. Conversely then, the symptoms of AIM appear to derive from the absolute CD8 expansion rather than from the virus infection *per se*.

## Materials and Methods

### Ethics statement

The Gambian Government/ MRC Laboratories Joint Ethics Committee approved this study. Participants were enrolled after individual written informed consent was obtained from the participant's parent/guardian. SCC 1206.

### Donors

This study was conducted in a peri-urban Medical Research Council (MRC) UK clinic, Sukuta, situated within the Government Sukuta Health Centre, serving a low-income population living in crowded conditions. A cohort of 120 children aged between 14 and 18 months were screened when they attended the local government health centre for their routine booster vaccination of diphtheria, tetanus, whole cell pertussis (DTwP) combined vaccine. All children were screened by enquiring about a maternal history of recent illness (e.g. fever) and a clinician examination for signs and symptoms of infectious mononucleosis, including weight and baseline observations (temperature, heart rate, weight and length). Any child found to be unwell (observations outside normal clinical range for age or maternal report of recent illness) or had a weight below that specified on the local Infant Welfare Card Growth Chart were not recruited into the study. (n = 6). Of the children not recruited, none of these showed clinical features suggestive of infectious mononucleosis. Five millilitres of blood was collected from each child into vacutainers containing EDTA (BD). A 500μl aliquot was removed and used to obtain a full blood count on each child using a M-series M16/M20 Haematology Analyser (Medonic, Sweden). A further 250μl aliquot was removed and whole blood flow cytometric staining performed. The remaining blood was layered on to 4mls of Lymphoprep (Axis-Shield, UK) in 15ml Leucosep tubes (Greiner Bio-One, UK). Following centrifugation, the plasma layer was removed, and cryopreserved in 2ml aliquots and stored at −70°C for downstream serology. The lymphocyte interphase was harvested and washed. Cells were counted and re-suspended in freezing medium (FCS (Sigma-Aldrich) supplemented with 10% (v/v) dimethyl sulfoxide (DMSO)) at approximately 5 x 10^6^/ml. Children were brought back one week later to receive the Pentavalent vaccination (DTwP, Hep B, Hib) (Easy Five Panacea Biotec). They were subsequently invited to return a week after vaccination and again at six months to undergo further blood sampling.

AIM patients were recruited from a cohort of young adults (18–25 years old) collected at the University of Birmingham, UK. All patients gave written informed consent to donate samples and experiments were approved by the South Birmingham Local Research Ethics Committee (reference number 07/Q2702/24). Patients were defined as AIM by having tonsillitis/sore throat, high lymphocyte counts and being heterophile antibody positive. Mononuclear cells were harvested from blood specimens and stored as described above.

### EBV genome loads

EBV genome loads were assayed by quantitative real-time PCR, as described elsewhere [46]. DNA extraction was performed from 1x10^6^ PBMCs using QIAmp DNA Blood Mini kit (Qiagen).

### Serology

IgG and IgM reactivity to EBV Viral Capsid Antigen (VCA) were measured using a previously described in-house immunofluorescence assay at the Institute for Cancer Studies, Birmingham [47,48] and the MRC-University of Glasgow Centre for Virus Research, University of Glasgow [49,50] respectively.

### Lymphocyte subset analysis by flow cytometric staining of human cells

For children a 100 μl volume of whole blood for each donor was stained with antibodies to the following surface markers: CD3 PE, CD4 PerCP, (BD Biosciences), CD8 efluor450 and CD27 APCalexafluor750 (Ebioscience) for 30 min at 4°C. Red blood cells were then lysed using 1:10 FACS Lysing Solution (BD Biosciences) and incubated for 10 min at room temperature. Cells were then washed twice in FACS buffer (PBS, 5% BSA, 5% EDTA) and re-suspended in Cytofix (BD, Biosciences). Samples were acquired on a Cyan ADP flow cytometer using Summit software (Beckman Coulter) at MRC Gambia.

Lymphocyte subsets from AIM patients were identified by staining with antibodies specific to: CD19 FITC, CD4 PE (Biolegend), CD27 APC elfluor 780, CD3 efluor 450 (eBioscience) and CD8 qDot 655 (Invitrogen). Samples were stained for 30 min on ice, washed and analysed immediately on an LSR-II flow cytometer (BD Biosciences). Data was analysed using Flow-Jo software (Treestar Inc).

### Cell surface and tetramer flow cytometric staining

Tetramers were used to identify and analyse the surface marker phenotype of epitope-specific CD8+ T-cells. From the aforementioned IFN-γ ELISPOT data we selected the following epitopes, B*0801 RAKFKQLL, B*3501 EPLPQGQLTAY and A*0201 GLCTLVAML for tetramer manufacture, as they were frequent targets of the immune response. Markers of activation (CD38 & HLDR), proliferation (Ki-67) and the anti-apoptotic marker, Bcl-2 were assessed. Tetramers were validated for specificity against HLA-matched and mismatched seropositive and seronegative donors. Tetramer staining was performed on cryopreserved PBMCs as described elsewhere. Cells were thawed and stained with LIVE/DEAD fixable Aqua Dead Cell Stain for 30 min at 4°C, washed and stained with 1μg of tetramer-PE for 15min at 37°C. Following two further washes, surface staining with CD3-Qdot655, CD4-Qdot605, CD8-Qdot705, CD14-V500, CD19-V500, CD38-APC and HLA-DR-Alexafluor700 were performed. Following fixing and permeabilisation as described above, intracellular staining with Ki67-Alexafluor488 and Bcl-2 (B-cell lymphoma 2)-V450 was performed. Fluorescence minus one samples were included to aid gating during subsequent flow cytometric analysis. A comparison of expression of the above phenotypic markers on EBV-specific and the total CD8+ T-cell populations were performed.

Compensation for fluorescence ‘spill-over’ was performed using the BD CompBead Anti-Mouse Ig set (BD Biosciences) and the antibodies described above. Briefly, antibodies were added to separate tubes containing one drop each of Anti-Mouse Ig beads and the negative control beads (which do not bind κ light chain-bearing immunoglobulin). Following a 30 min incubation at 4°C, beads were washed and re-suspended in FACS buffer.

### Statistics

All statistical analyses were performed using Graphpad Prism version 5.0 for Macintosh (GraphPad Software, San Diego California, USA, www.graphpad.com). Comparisons between variables were performed using the Mann-Whitney U test (two-tailed) and for non-parametrically distributed data, the Wilcoxon matched pairs test (for comparisons between total CD8+ and virus-specific CD8+ T-cells made within individuals) was used. Correlations between non-normally distributed data were made using the Spearman’s rank correlation coefficient.

## Supporting Information

S1 Fig Comparison of plasma IgG VCA antibody titre in children assayed at visits one and four and relation to virus load. IgG VCA titres were assessed from paired plasma samples collected from 26 children at visit one and four (A). From these children’s samples, IgG VCA titres and virus loads determined from (B) visit one and (C) visit four were plotted against each other.
(TIF)

S2 Fig CD4:CD8 ratios in IgM-IgG+ Gambian children at visit one and six months later at visit four. (TIFF)

S1 Table Age, sex, haemoglobin, weight and cell counts in Gambian children participating in the study and drop-outs. (TIFF)
