# Supplementary figures and images for: Early Virological and Immunological Events in Asymptomatic Epstein-Barr Virus Infection in African Children
Source: PLoS Pathog. 2015 Mar 27;11(3):e1004746. doi: 10.1371/journal.ppat.1004746 (PMC4376400; doi:10.1371/journal.ppat.1004746)

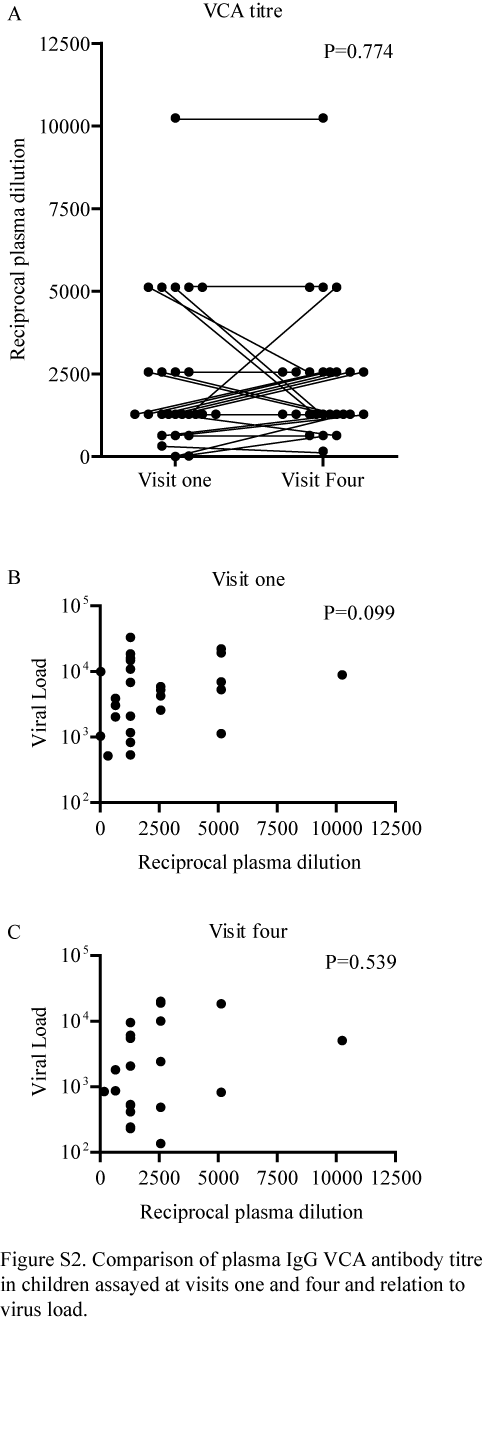

Supplement: S1 Fig — IgG VCA titres were assessed from paired plasma samples collected from 26 children at visit one and four (A). From these children’s samples, IgG VCA titres and virus loads determined from (B) visit one and (C) visit four were plotted against each other. (TIF) [file ppat.1004746.s001.tif]

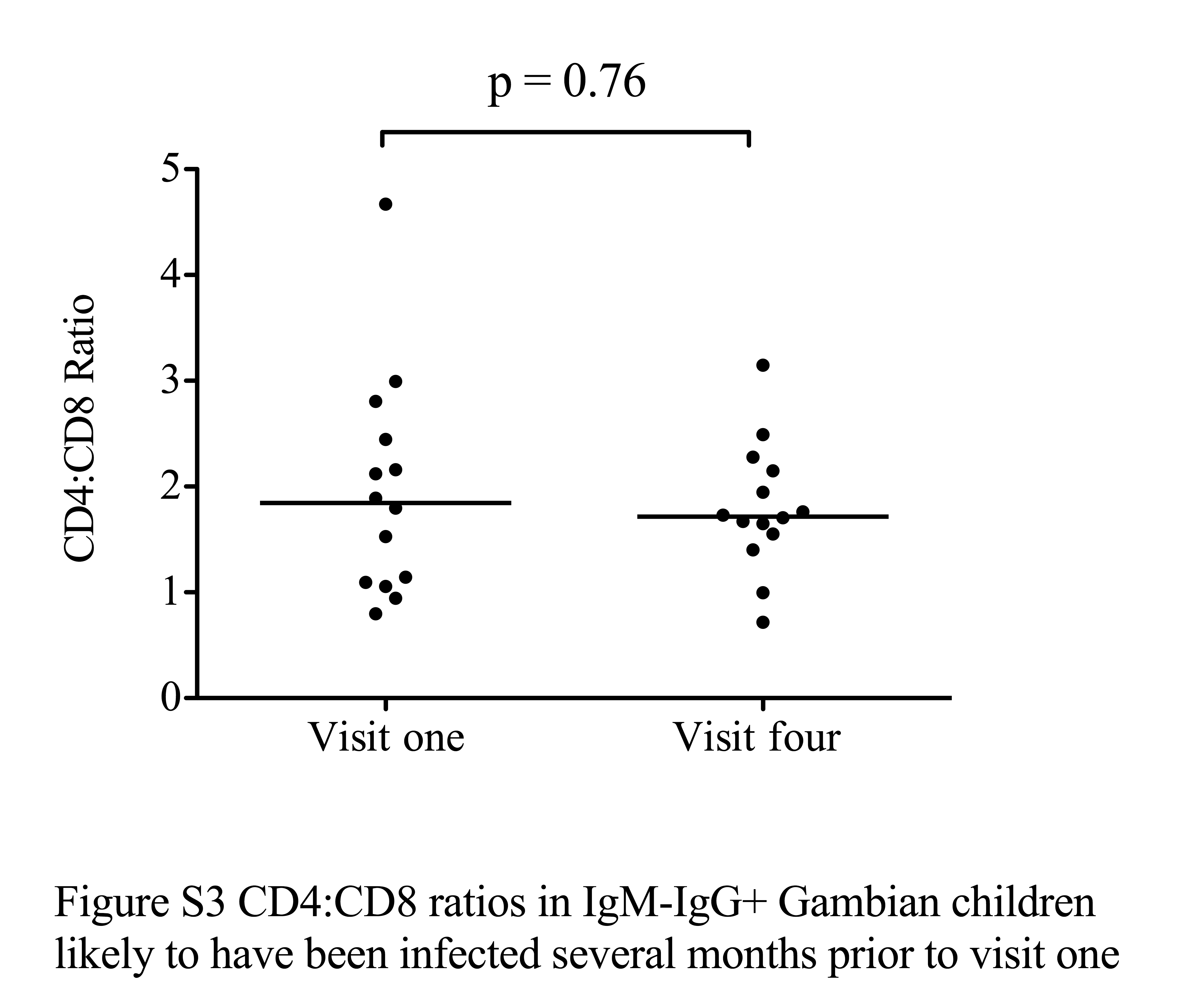

Supplement: S2 Fig — (TIFF) [file ppat.1004746.s002.tiff]

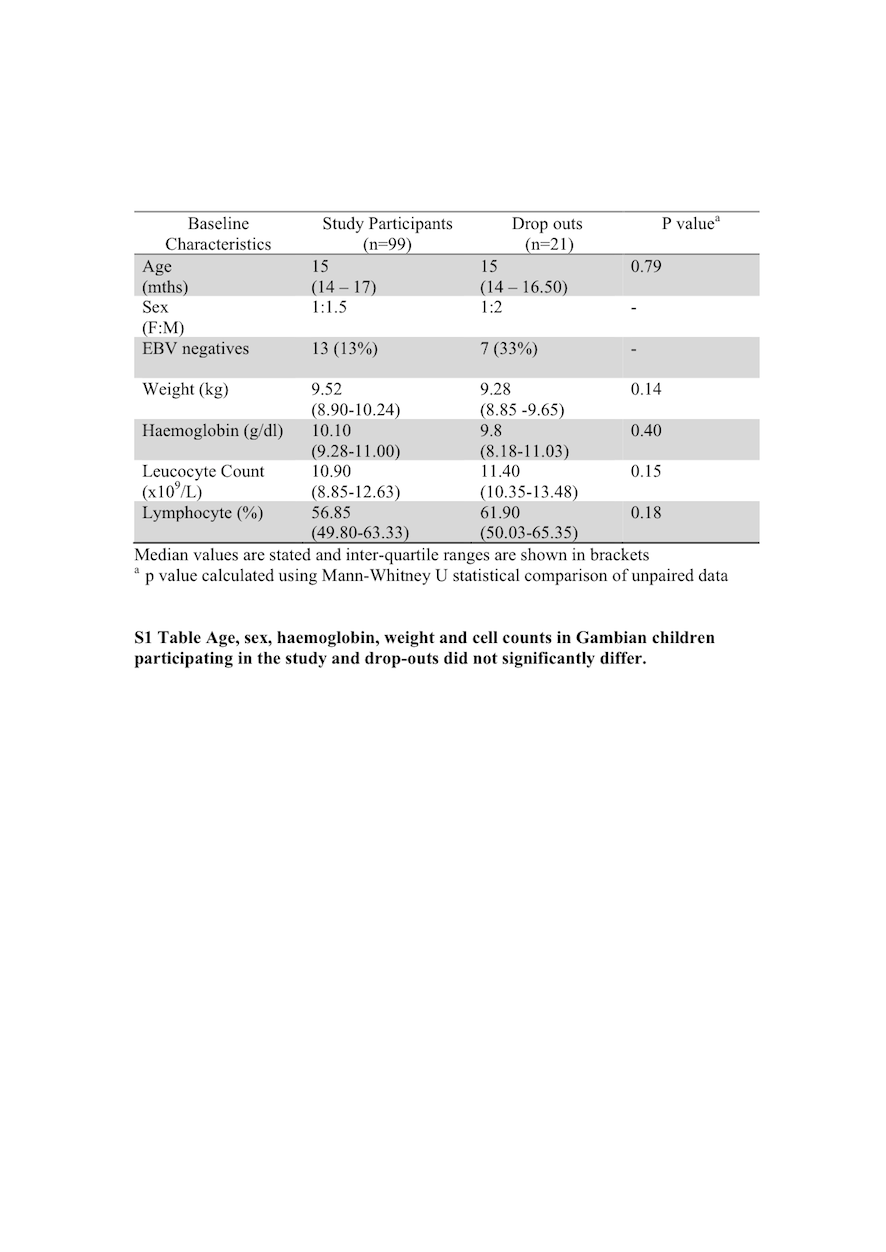

Supplement: S1 Table — (TIFF) [file ppat.1004746.s003.tiff]
